# Maternal disease factors associated with neonatal jaundice: a case–control study

**DOI:** 10.1186/s12884-022-04566-6

**Published:** 2022-03-24

**Authors:** Youngjae Yu, Jinwha Choi, Myeong Hoon Lee, KangHyun Kim, Hyun Mee Ryu, Hyun Wook Han

**Affiliations:** 1 Department of Biomedical Informatics, CHA University School of Medicine, CHA University, Seongnam, Korea; 2 Institute for Biomedical Informatics, CHA University School of Medicine, CHA University, Seongnam, Republic of Korea; 3 Department of Pediatrics, Korea University College of Medicine, Seoul, Korea; 4 Department of Obstetrics and Gynecology, CHA Bundang Medical Center, CHA University School of Medicine, Seongnam, Korea

**Keywords:** Maternal disease factors, Neonatal jaundice, Prevention, Prediction, Disease network, National Health Insurance Service–National Sample Cohort

## Abstract

**Background:**

Neonatal jaundice is common, and despite the considerable medical costs associated with it, there are still few studies on the maternal factors associated with it. Identification of maternal factors associated with neonatal jaundice is very important in terms of prevention, screening and management of neonatal jaundice. The current study aimed to identify maternal disease factors associated with neonatal jaundice.

**Methods:**

We compared the maternal disease diagnostic codes during pregnancy (study A) and 1 year before conception (study B) in mothers whose insurance claims data included newborns treated for neonatal jaundice before birth registration via the National Health Insurance Service–National Sample Cohort (control group). To decrease the effect of confounding variables, the neonatal jaundice and control groups were matched at a ratio of 1:10 via propensity score matching using covariates including age and income.

**Results:**

The matched samples for studies A and B included 4,026 and 3,278 (jaundice group: 366 and 298) delivery cases, respectively. In both studies, the jaundice group had a higher proportion of patients who underwent cesarean section than the control group. In study A, other diseases of the digestive system had the highest odds ratio (OR) (K92; adjusted OR: 14.12, 95% confidence interval [CI]: 2.70–82.26). Meanwhile, gastritis and duodenitis had the lowest OR (K29; adjusted OR: 0.39, 95% CI: 0.22–0.69). In study B, salpingitis and oophoritis had the highest OR (N70; adjusted OR: 3.33, 95% CI: 1.59–6.94). Heartburn had the lowest OR (R12; adjusted OR: 0.29, 95% CI:0.12–0.71).

**Conclusions:**

This study identified maternal disease factors correlated with neonatal jaundice during pregnancy and 1 year before conception. Maternal risk factors for neonatal jaundice included syphilis and leiomyoma during pregnancy, and salpingo-oophoritis before pregnancy. The protective factors included infection, inflammatory diseases, and dyspepsia.

## Backgrounds 

Neonatal jaundice is a common disease [1]. In Korea, it is the most common cause of admission among newborns [2], and medical expenses correlated with jaundice exceeded $10 million in 2012 [3]. Moreover, it still poses global burden particularly in low- and middle-income countries where the immediate assessment of serum bilirubin concentration is challenging and treatment is often delayed [4, 5]. Moreover, recent studies have reported that neonatal jaundice may be a risk factor for pediatric diseases such as asthma [6, 7], autism spectrum disorders [8, 9], attention deficit hyperactivity disorder (ADHD) [10], and epilepsy [11]. Therefore, identifying maternal risk factors for neonatal jaundice is important in providing cost-effective healthcare expenditure and predicting jaundice-associated diseases.

However, recent studies have not assessed these factors and only a few predisposing factors, including maternal age, race, primiparity, teenage pregnancy, diabetes mellitus, Rh incompatibility, ABO incompatibility, oxytocin use during labor, and breastfeeding, were identified [4, 12]. These factors were demographic or pregnancy-related, and there has been no study to identify risk factors for neonatal jaundice related to the mother's own disease.

Many studies so far were cross-sectional studies [13, 14], and nation-wide study focused on neonatal jaundice requiring the treatment in clinical situation has not been done well. In Korea, all citizens are covered by the National Health Insurance [15], and a database for claims data has been established [16]. Moreover, the antenatal care (ANC) coverage of married women approaches about 100%, and the average number of antenatal care visits is over 13 times [17]. Based on the high ANC coverage and the longitudinal data on individuals, the study on the gestation or antenatal period can be conducted appropriately. Hence, the current study aimed to analyze the maternal disease risk factors for neonatal jaundice during pregnancy and 1 year before conception using data from the National Health Insurance claims database.

## Methods

### Data source and variables

The National Health Insurance Service–National Sample Cohort (NHIS-NSC) established by the National Health Insurance Service in South Korea was used [16]. This database (DB) is a representative sample that randomly selected 1 million people, accounting for about 2.2% of the Korean population in 2002. Moreover, it contains sample data obtained from 2002 to 2013 [18]. In this study, the qualification DB and treatment DB of the NHIS-NSC were used. Five variables of the qualification DB (patient ID, sex, year, age, and income rank), five variables of statement data (patient ID, claim number, visit date, principal diagnosis, and additional diagnosis), and three variables of type of disease data (claim number, visit date, and diagnosis) in the treatment DB were utilized. Age is divided into 19 groups from 0 to 85 years old at 5-year intervals (age 0, 1–4, 5–9, …, and over 84). As the age of participants considered in this study was 15–49 years old, it was regrouped subsequently into three groups with ages 15–24, 25–34, and 35–49. The income rank is divided into 11 groups at deciles with medical aid beneficiaries, and it was regrouped into 5 groups at 20% intervals. Variables about diagnosis were distinguished using the Korean Standard Classification of Diseases, version 6 (KCD-6), which is the Korean modified version of the 10th revision of the International Statistical Classification of Diseases and Related Health Problems (ICD-10).

All methods were carried out in accordance with relevant guidelines and regulations.

### Data preprocessing and case selection

The principal diagnosis and additional diagnosis of statement data were integrated into one diagnostic variable and were then merged with the type of disease data according to claim number. Based on the merged diagnosis data, details regarding delivery and age of the participants (15–49 years old) at the year of delivery date were extracted. Then, the pregnancy records of patients who had data about delivery were collected. In cases in which treatment for neonatal jaundice were provided before birth registration, when insurance claims were made by the mother, the diagnosis of neonatal jaundice is included in the mother’s record. Hence, these cases were included in the jaundice group. Cases with diagnostic codes correlated with neonatal jaundice within 4 weeks after the delivery date were included in the jaundice group. Meanwhile, the control group included cases in which the diagnosis of neonatal jaundice was not attached to the mother.

In this study, KCD-6 codes related to delivery [19–21], pregnancy [19, 22, 23] and neonatal jaundice were selected to identify each event. Preterm delivery and multiple gestation were defined as at least one record of the related codes within 4 weeks before and after the delivery date.

As the NHIS-NSC included a sample established from the claims data, but not designed for the study, the diagnoses entered in the records did not always indicate new-onset diseases. The same diagnosis codes might have been recorded repeatedly. In such a case, it was counted as one. If the same person delivered several times, each delivery was considered an independent case. The minimum interval from delivery to diagnosis of the next pregnancy was 4 weeks. Considering that periviable birth is defined as delivery during at least 20 weeks of gestation [24], the minimum interval from the previous to the next delivery was 24 weeks. The maximum duration from the diagnosis of pregnancy to delivery was 44 weeks [25]. After cross-joining pregnancy and delivery records, the joint records were listed chronologically. The date of pregnancy diagnosis was defined as the visit date of the first pregnancy record among all pregnancy records. Cases with diagnostic codes related to abortion (O00-O08, pregnancy with abortive outcome) [22, 23, 26] or stillbirths (O36.4, maternal care for intrauterine death; Z37.1, single stillbirth; Z37.4, twins, both stillborn; and Z37.7, other multiple births, all stillborn) [19, 22, 26] up to 4 weeks after the delivery date were excluded. Deliveries assigned with codes including O82 and O84.2 (multiple delivery, all via cesarean section) were considered as cesarean section. Further, deliveries assigned with codes such as O80, O81, O83, and O84.0 (multiple delivery, all spontaneous) and O84.1 (multiple delivery, all using forceps and vacuum extractor) were considered as vaginal. If the two types of delivery were present, cesarean section (O82, O84.2) was prioritized. Cases in which the type of delivery was not identified were excluded.

In this study, two studies, study A and B, were conducted. One was about diseases during ANC (study A), and the second was about diseases 1 year before ANC (study B). In study A, claims data from each pregnancy diagnosis date to day 1 before the delivery date were extracted. Cases that had no record in the ANC period, other than diagnostic codes correlated with pregnancy, were excluded to identify possible maternal risk factors. In study B, claims data from 1 year before each pregnancy diagnosis date to day 1 before the pregnancy diagnosis date were extracted. In the analyses of both two studies, the only first three characters of the diagnosis codes were used.

### Statistical analysis

The two-sided Fisher’s exact test with 95% CI for the categorical variables was performed. The *t*-test was used to assess continuous variables. To decrease the effect of confounding variables, the jaundice and control groups were matched at a ratio of 1:10 via propensity score matching (PSM) with nearest neighbor matching. Age at the time of delivery and income at the time of pregnancy diagnosis were considered covariates. *MatchIt* package [27] was used to perform PSM. The results obtained by repeating PSM 1,000 times by randomly shuffling the order of records were used for the analysis of matched samples. The average number of cases, odds ratio, and *p*-value were calculated only for significant findings from the 1,000 results obtained using PSM. If the odds ratio was infinite, it was excluded from the average. Diseases that have more than 900 significant results, with a mean odds ratio of > 1 and a mean *p*-value of < 0.05, were considered a risk factor. Moreover, those with a mean odds ratio of < 1 and a mean *p*-value of < 0.05 were considered a protective factor. Conditional logistic regression analyses, adjusted for preterm delivery, delivery mode, multiple gestation and ANC duration, were performed for the diseases which have more than 900 significant results in the univariable analyses. *Survival* package [28] was used to perform conditional logistic regression analyses.

Results with a lower bound of > 1 or an upper bound of < 1 and a *p*-value of < 0.05 were considered significant. *igraph* package [29] was used to make a network image for the identified risk/protective factors. R (version 3.6.2) [30] was used in all analyses.

## Results

### Demographic characteristics of the participants

**Fig. 1:**
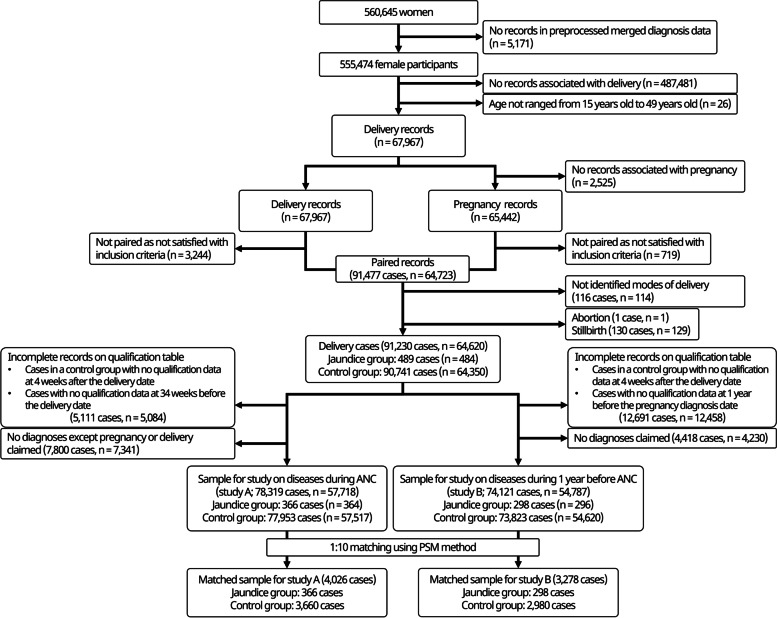
Flowchart of the inclusion and exclusion process. ANC, antenatal care; PSM, propensity score matching

Figure 1 shows the flowchart of the case selection process. 555,474 of 560,645 women, included in NHIS-NSC from 2002 to 2013, had significant claims data about diagnoses. 67,967 of those 555,474 women had claims data related to diagnosis of delivery and were 15 to 49 years old at the time of the delivery. 65,442 participants of them, who had delivery records, had claims data related to the diagnosis of pregnancy. With pairing the delivery and pregnancy records, 91,477 delivery cases (64,723 women), satisfied with the time intervals which were defined as inclusion criteria in this study, were identified. 116 cases of 91,477 delivery cases were excluded as the delivery modes were not identified, and 131 cases of abortion or stillbirth were excluded subsequently. The participants with several delivery cases were included in each step unless all cases were excluded. Among 91,230 delivery cases, 5,111 cases incomplete on qualification DB around the gestation period and 7,800 cases that had no diagnosis records except pregnancy or delivery during ANC were excluded to consist of the sample for study A. For study B, 12,691 incomplete cases and 4,418 cases with no diagnosis records during ANC were excluded.

The sample in study A included 78,319 cases (*n* = 57,718). Among them, 366 cases (*n* = 364) were included in the jaundice group and 77,953 cases (*n* = 57,517) in the control group. The sample in study B had 74,121 cases (*n* = 54,787). Among them, 298 cases (*n* = 296) were included in the jaundice group and 73,823 cases (*n* = 54,620) in the control group. The *n* value indicated the number of mothers, not delivery cases. If a mother has delivered several times, it can be included in both the jaundice and control groups. Thus, the total number of patients in the jaundice and control groups did not correspond to the total population. The jaundice group accounted for 0.47% (366 in 78,319 cases) and 0.40% (298 in 74,121 cases) of all delivery cases in studies A and B, respectively. There was a significant difference in terms of income at the time of pregnancy diagnosis, multiple gestation, and ANC duration between the two groups in study A, but not in study B. However, the Cochran–Armitage trend test result (chi-square test for trend in proportion) for income was significant in studies A and B (*p*-value = 0.002 and 0.010, respectively). There was a significant difference in the mode of delivery between the two groups in studies A and B (Table 1).

**Table 1:** Demographic data of the unmatched samples

Characteristics	Study A (78,319 cases)			Study B (74,121 cases)		
	Jaundice group (366 cases)	Control group (77,953 cases)	*p*-value	Jaundice group (298 cases)	Control group (73,823 cases)	*p*-value
Age^a^ (years)			0.305			0.147
15–24	20 (5.5)	3,566 (4.6)		12 (4.0)	3,232 (4.4)	
25–34	294 (80.3)	61,240 (78.6)		247 (82.9)	57,851 (78.4)	
35–49	52 (14.2)	13,147 (16.9)		39 (13.1)	12,740 (17.3)	
Income^b^			0.005^*^			0.054
1 (lowest)	25 (6.8)	8,221 (10.5)		20 (6.7)	7,645 (10.4)	
2	48 (13.1)	12,553 (16.1)		41 (13.8)	11,627 (15.7)	
3	107 (29.2)	20,567 (26.4)		84 (28.2)	19,465 (26.4)	
4	107 (29.2)	24,046 (30.8)		90 (30.2)	23,065 (31.2)	
5 (highest)	79 (21.6)	12,566 (16.1)		63 (21.1)	12,021 (16.3)	
Preterm delivery	5 (1.4)	1,407 (1.8)	0.693	2 (0.7)	1,328 (1.8)	0.187
Cesarean section	181 (49.5)	29,734 (38.1)	< 0.001^*^	138 (46.3)	27,998 (37.9)	< 0.001^*^
Multiple gestation	11 (3.0)	1,156 (1.5)	0.027^*^	7 (2.3)	1,073 (1.5)	0.215
ANC duration	211.50 ± 48.33	222.20 ± 35.94	< 0.001^*^	217.21 ± 41.29	221.75 ± 37.27	0.059

The number and ratio of cases in each group were presented as N (%), except for the duration of ANC that was expressed as mean ± standard deviation days

*ANC* Antenatal care

^*^Significance at *p*-value of < 0.05

^a^age at the time of delivery, ^b^ income at the time of pregnancy diagnosis

The matched sample for study A had 4,026 cases (jaundice group: 366, control group: 3,660), and that for study B had 3,278 cases (jaundice group: 298, control group: 2,980). All the 1,000 matched samples significantly differed in terms of the type of delivery in both two studies (Table 2). There was also a significant difference in ANC duration in all the 1,000 matched samples of study A.

**Table 2:** Demographic data of the matched samples

Characteristics	Study A (4,026 cases)			Study B (3,278 cases)		
	Jaundice group (366 cases)	Control group (3,660 cases)	*p*-value	Jaundice group (298 cases)	Control group (2,980 cases)	*p*-value
Preterm delivery	5 (1.4)	66.00 (1.8)	0.732	2 (0.7)	52.17 (1.8)	0.251
Cesarean section	181 (49.5)	1,376.70 (37.6)	< 0.001^*^	138 (46.3)	1,109.80 (37.2)	< 0.001^*^
Multiple gestation	11 (3.0)	53.44 (1.5)	0.047^*a^	7 (2.3)	42.85 (1.4)	0.251^b^
ANC duration	211.50 ± 48.33	222.31 ± 36.01	< 0.001^*^	217.21 ± 41.29	222.16 ± 37.00	0.055^c^

The number and ratio of cases in each group were presented as N (%), except for the duration of ANC that was expressed as mean ± standard deviation days. The average number of cases and mean *p*-values from 1,000 matched samples were presented

*ANC* Antenatal care

^*^ Significance at *p*-value < 0.05

^a^*P*-value from the 670 matched samples were significant, with a mean of 0.027

^b^*P*-value from the 15 matched samples were significant, with a mean of 0.031

^c^*P*-value from the 541 matched samples were significant, with a mean of 0.032

### Odds ratios for each diagnosis code in the unmatched samples

Tables 3 and 4 show the unadjusted odds ratios for neonatal jaundice according to disease that showed significant results in unmatched samples for studies A and B, respectively.

**Table 3:** Maternal diseases during antenatal care and their unadjusted odds ratio for neonatal jaundice obtained from the unmatched samples (study A)

Disease (diagnosis code)	Jaundice group (366 cases)	Control group (77,953 cases)	Unadjusted odds ratio (95% CI)	*p*-value^*^
Obstetrical tetanus (A34)	1 (0.3)	1 (0)	212.33 (2.71–14,121.54)	0.009
Other diseases of digestive system (K92)	4 (1.1)	86 (0.1)	10.00 (2.65–26.79)	< 0.001
Alopecia areata (L63)	2 (0.5)	46 (0.1)	9.31 (1.09–35.79)	0.021
Other surgical follow-up care (Z48)	6 (1.6)	210 (0.3)	6.17 (2.22–13.79)	< 0.001
Other and unspecified syphilis (A53)	8 (2.2)	370 (0.5)	4.69 (1.99–9.43)	< 0.001
Leiomyoma of uterus (D25)	11 (3.0)	769 (1.0)	3.11 (1.53–5.66)	0.001
Infections of genitourinary tract in pregnancy (O23)	29 (7.9)	9,033 (11.6)	0.66 (0.43–0.96)	0.027
Acute nasopharyngitis (J00)	22 (6.0)	7,442 (9.5)	0.61 (0.37–0.93)	0.020
Acute upper respiratory infections of multiple and unspecified sites (J06)	20 (5.5)	7,041 (9.0)	0.58 (0.35–0.91)	0.017
Vasomotor and allergic rhinitis (J30)	21 (5.7)	7,593 (9.7)	0.56 (0.34–0.88)	0.008
Other functional intestinal disorders (K59)	9 (2.5)	3,755 (4.8)	0.50 (0.23–0.96)	0.036
Acute tonsillitis (J03)	11 (3.0)	4,773 (6.1)	0.48 (0.23–0.86)	0.011
Acute bronchitis (J20)	12 (3.3)	5,547 (7.1)	0.44 (0.23–0.78)	0.003
Gastritis and duodenitis (K29)	13 (3.6)	7,014 (9.0)	0.37 (0.20–0.65)	< 0.001
Dyspepsia (K30)	4 (1.1)	2,491 (3.2)	0.33 (0.09–0.87)	0.016
Fever of other and unknown origin (R50)	2 (0.5)	1,820 (2.3)	0.23 (0.03–0.84)	0.015

The number and ratio of cases in each group are presented as N (%)

^*^Significance at *p*-value < 0.05

*CI* Confidence interval

**Table 4:** Maternal diseases during 1 year before antenatal care and their unadjusted odds ratio for neonatal jaundice obtained from the unmatched samples (study B)

Disease (diagnosis code)	Jaundice group (298 cases)	Control group (73,823 cases)	Unadjusted odds ratio (95% CI)	*p*-value^*^
Polyarteritis nodosa and related conditions (M30)	1 (0.3)	0 (0)	Inf (6.35-Inf)	0.004
Disorders of glycoprotein metabolism (E77)	1 (0.3)	1 (0)	248.76 (3.16–15,989.22)	0.008
Acute and transient psychotic disorders (F23)	1 (0.3)	4 (0)	62.11 (1.26–640.90)	0.020
Other disorders of skin and subcutaneous tissue in diseases classified elsewhere (L99)	1 (0.3)	4 (0)	62.11 (1.26–640.90)	0.020
Family history of malignant neoplasm (Z80)	1 (0.3)	4 (0)	62.11 (1.26–640.90)	0.020
Paroxysmal tachycardia (I47)	3 (1.0)	73 (0.1)	10.28 (2.06–31.51)	0.004
Acute posthemorrhagic anemia (D62)	3 (1.0)	105 (0.1)	7.14 (1.44–21.64)	0.010
Respiratory tuberculosis, not confirmed bacteriologically or histologically (A16)	4 (1.3)	154 (0.2)	6.51 (1.74–17.19)	0.004
Other disorders of pigmentation (L81)	3 (1.0)	148 (0.2)	5.06 (1.03–15.23)	0.023
Alopecia areata (L63)	5 (1.7)	298 (0.4)	4.21 (1.35–10.05)	0.008
Other superficial mycoses (B36)	5 (1.7)	310 (0.4)	4.05 (1.30–9.65)	0.009
Anogenital herpes viral infection (A60)	7 (2.3)	541 (0.7)	3.26 (1.29–6.85)	0.007
Salpingitis and oophoritis (N70)	10 (3.4)	785 (1.1)	3.23 (1.53–6.06)	0.002
Urethritis and urethral syndrome (N34)	7 (2.3)	554 (0.8)	3.18 (1.26–6.68)	0.008
Eustachian salpingitis and obstruction (H68)	8 (2.7)	794 (1.1)	2.54 (1.08–5.09)	0.017
Acute bronchitis (J20)	80 (26.8)	23,856 (32.3)	0.77 (0.59–1.00)	0.047
Gastritis and duodenitis (K29)	111 (37.2)	33,027 (44.7)	0.73 (0.57–0.93)	0.010
Vasomotor and allergic rhinitis (J30)	92 (30.9)	29,922 (40.5)	0.66 (0.51–0.84)	< 0.001
Dyspepsia (K30)	35 (11.7)	14,324 (19.4)	0.55 (0.38–0.79)	< 0.001
Other disorders of nose and nasal sinuses (J34)	7 (2.3)	4,184 (5.7)	0.40 (0.16–0.84)	0.011
Other disorders of breast (N64)	3 (1.0)	2,221 (3.0)	0.33 (0.07–0.97)	0.040
Heartburn (R12)	5 (1.7)	4,209 (5.7)	0.28 (0.09–0.67)	< 0.001
Pain associated with micturition (R30)	0 (0)	990 (1.3)	0 (0–0.92)	0.038

The number and ratio of cases in each group were expressed as N (%)

^*^Significance at *p*-value < 0.05

*CI* Confidence interval

In study A, obstetrical tetanus (A34) had the largest OR (212.33, 95% CI: 2.71–14,121.54). Fever of other and unknown origin (R50) had the lowest OR (0.23, 95% CI: 0.03–0.84).

In study B, polyarteritis nodosa and related conditions (M30) had the largest OR (95% CI: 6.35-infinite). However, there was only case in the jaundice group. Pain associated with micturition (R30) had the lowest OR (0, 95% CI: 0–0.92).

Acute bronchitis (J20), vasomotor and allergic rhinitis (J30), gastritis and duodenitis (K29), dyspepsia (K30), and alopecia areata (L63) showed significance in the unmatched samples of the two studies. Among them, the OR of alopecia areata (L63) was > 1.

### Risk and protective factors in the matched samples

**Fig. 2:**
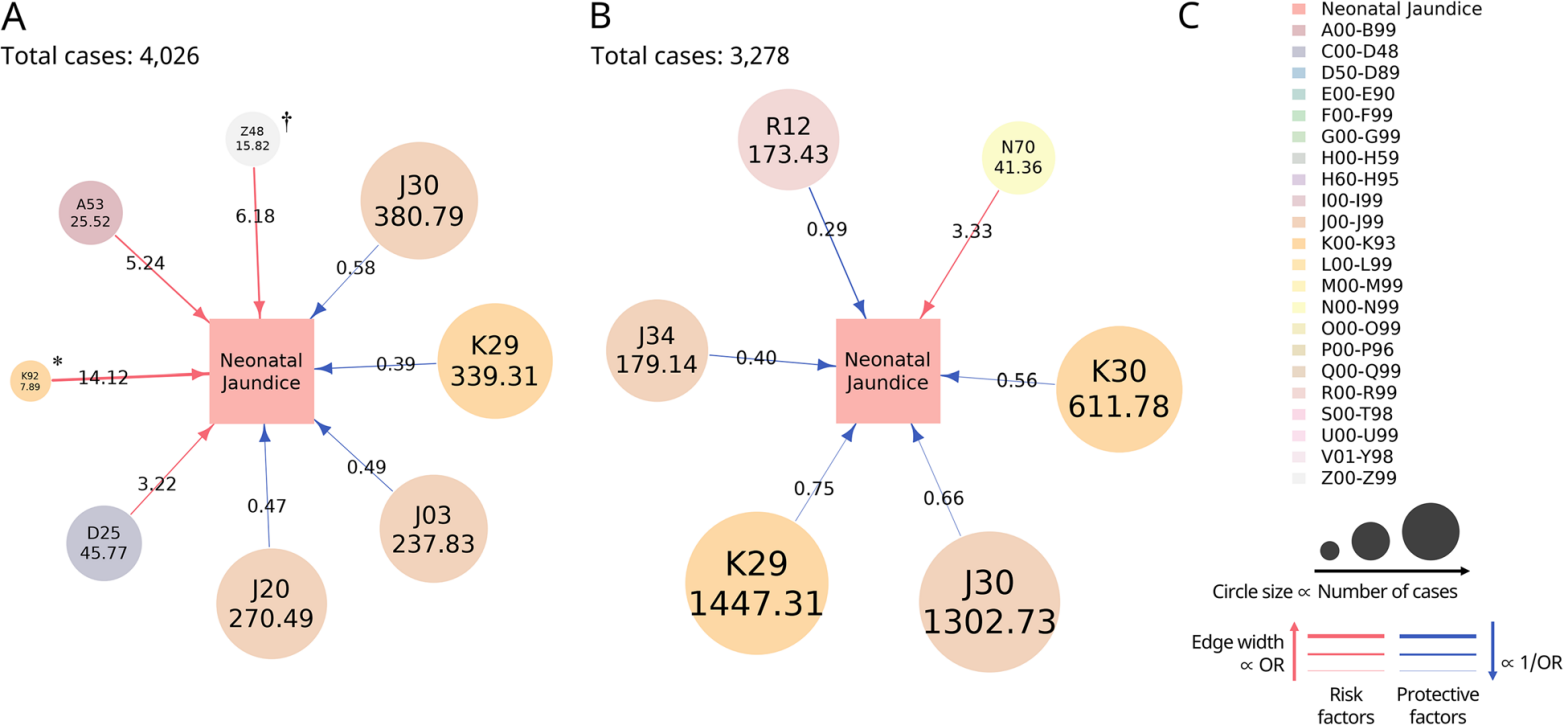
Disease network image about maternal risk factors and protective factors for neonatal jaundice**. A** Maternal diseases during ANC associated with neonatal jaundice (Study A). **B** Maternal diseases during 1 year before ANC associated with neonatal jaundice (Study B). **C** Index. Risk factors are illustrated as red lines and protective factors as blue lines. The average odds ratio is represented by number on the line and the average number of cases as the number in the circle. The major classification of diagnosis codes is represented in a different color. The circle size is proportional to the number of cases. The edge width is proportional to the odds ratio in the case of risk factors and inversely proportional to the odds ratio in the case of protective factors. ANC, antenatal care; A53, other and unspecified syphilis; D25, leiomyoma of the uterus; D62, acute posthemorrhagic anemia; J03, acute tonsillitis; J20, acute bronchitis; J30, vasomotor and allergic rhinitis; J34, other disorders of the nose and nasal sinuses; K29, gastritis and duodenitis; K30, dyspepsia; K92, other diseases of the digestive system; N70, salpingitis and oophoritis; R12, heartburn; Z48, other surgical follow-up care. ^*^ K92, 7.89; ^†^ Z48, 15.82

For diseases that showed significance more than 900 times in 1000 times of PSM, the average number of cases and average odds ratio are depicted in Fig. 2 (network image) and Table 5. Adjusted ORs were calculated for the diseases which have more than 900 significant results in the univariable analyses, as the primary outcome of this study.

**Table 5:** Maternal diseases associated with neonatal jaundice identified from the matched samples in studies A and B

	Univariable				Multivariable						
Disease (diagnosis code)	Jaundice group	Control group	Unadjusted odds ratio (95% CI)	*p*-value^*^	Jaundice group	Control group	Adjusted odds ratio (95% CI) [*p*-value^*^]				
							Disease	Preterm	C-section	Multiple gestation	ANC duration
***Study A***	366 cases	3,660 cases			366 cases	3,660 cases					
Other diseases of digestive system (K92)	4 (1.1)	3.80 (0.1)	13.97 (2.21–238.30)	0.004	4 (1.1)	3.89 (0.1)	14.12 (2.70–82.26) [0.003^*^]	0.54 (0.21–1.38) [0.208]	1.55 (1.24–1.93) [< 0.001^*^]	1.60 (0.79–3.21) [0.234]	0.99 (0.99–1.00) [< 0.001^*^]
Other surgical follow-up care (Z48)	6 (1.6)	9.82 (0.3)	6.96 (1.97–24.24)	0.003	6 (1.6)	9.82 (0.3)	6.18 (2.09–18.53) [0.003^*^]	0.53 (0.21–1.37) [0.200]	1.51 (1.21–1.89) [< 0.001^*^]	1.71 (0.86–3.40) [0.166]	0.99 (0.99–1.00) [< 0.001^*^]
Other and unspecified syphilis (A53)	8 (2.2)	17.52 (0.5)	4.94 (1.81–12.47)	0.002	8 (2.2)	17.52 (0.5)	5.24 (2.18–12.61) [< 0.001^*^]	0.53 (0.21–1.37) [0.201]	1.56 (1.25–1.95) [< 0.001^*^]	1.69 (0.85–3.36) [0.177]	0.99 (0.99–1.00) [< 0.001^*^]
Leiomyoma of uterus (D25)	11 (3.0)	34.75 (0.9)	3.32 (1.50–6.80)	0.003	11 (3.0)	34.77 (1.0)	3.22 (1.59–6.52) [0.003^*^]	0.53 (0.21–1.36) [0.198]	1.54 (1.23–1.92) [< 0.001^*^]	1.66 (0.83–3.30) [0.194]	0.99 (0.99–1.00) [< 0.001^*^]
Vasomotor and allergic rhinitis (J30)	21 (5.7)	357.13 (9.8)	0.56 (0.34–0.89)	0.014	21 (5.7)	359.79 (9.8)	0.58 (0.37–0.92) [0.024^*^]	0.54 (0.21–1.38) [0.207]	1.55 (1.24–1.94) [< 0.001^*^]	1.67 (0.84–3.32) [0.187]	0.99 (0.99–1.00) [< 0.001^*^]
Acute tonsillitis (J03)	11 (3.0)	224.56 (6.1)	0.48 (0.23–0.88)	0.016	11 (3.0)	226.83 (6.2)	0.49 (0.26–0.91) [0.025^*^]	0.53 (0.21–1.36) [0.197]	1.55 (1.24–1.94) [< 0.001^*^]	1.67 (0.84–3.32) [0.186]	0.99 (0.99–1.00) [< 0.001^*^]
Acute bronchitis (J20)	12 (3.3)	258.30 (7.1)	0.45 (0.23–0.81)	0.006	12 (3.3)	258.49 (7.1)	0.47 (0.26–0.84) [0.013^*^]	0.53 (0.21–1.36) [0.198]	1.55 (1.24–1.94) [< 0.001^*^]	1.67 (0.84–3.31) [0.187]	0.99 (0.99–1.00) [< 0.001^*^]
Gastritis and duodenitis (K29)	13 (3.6)	326.31 (8.9)	0.38 (0.20–0.66)	< 0.001	13 (3.6)	326.31 (8.9)	0.39 (0.22–0.69) [0.001^*^]	0.53 (0.21–1.36) [0.199]	1.55 (1.24–1.94) [< 0.001^*^]	1.76 (0.88–3.50) [0.149]	0.99 (0.99–1.00) [< 0.001^*^]
***Study B***	298 cases	2,980 cases			298 cases	2,980 cases					
Salpingitis and oophoritis (N70)	10 (3.4)	31.30 (1.1)	3.37 (1.45–7.20)	0.005	10 (3.4)	31.36 (1.1)	3.33 (1.59–6.94) [0.003^*^]	0.30 (0.07–1.27) [0.106]	1.42 (1.11–1.82) [0.007^*^]	1.47 (0.63–3.44) [0.421]	1.00 (0.99–1.00) [0.051]
Gastritis and duodenitis (K29)	111 (37.2)	1,332.70 (44.7)	0.73 (0.57–0.94)	0.017	111 (37.2)	1,336.31 (44.8)	0.75 (0.58–0.95) [0.023^*^]	0.30 (0.07–1.28) [0.108]	1.43 (1.12–1.83) [0.006^*^]	1.53 (0.66–3.55) [0.372]	1.00 (0.99–1.00) [0.092]
Vasomotor and allergic rhinitis (J30)	92 (30.9)	1,210.73 (40.6)	0.65 (0.50–0.85)	0.002	92 (30.9)	1,210.73 (40.6)	0.66 (0.51–0.86) [0.003^*^]	0.30 (0.07–1.27) [0.107]	1.43 (1.12–1.83) [0.006^*^]	1.53 (0.66–3.54) [0.376]	1.00 (0.99–1.00) [0.102]
Dyspepsia (K30)	35 (11.7)	576.78 (19.4)	0.56 (0.37–0.80)	0.001	35 (11.7)	576.78 (19.4)	0.56 (0.39–0.81) [0.003^*^]	0.30 (0.07–1.28) [0.109]	1.43 (1.12–1.83) [0.006^*^]	1.57 (0.68–3.64) [0.347]	1.00 (0.99–1.00) [0.093]
Other disorders of nose and nasal sinuses (J34)	7 (2.3)	171.37 (5.8)	0.40 (0.16–0.85)	0.014	7 (2.3)	172.14 (5.8)	0.40 (0.19–0.87) [0.022^*^]	0.30 (0.07–1.26) [0.104]	1.42 (1.11–1.81) [0.007^*^]	1.55 (0.67–3.59) [0.358]	1.00 (0.99–1.00) [0.064]
Heartburn (R12)	5 (1.7)	168.43 (5.7)	0.29 (0.09–0.69)	0.002	5 (1.7)	168.43 (5.7)	0.29 (0.12–0.71) [0.008^*^]	0.30 (0.07–1.28) [0.108]	1.43 (1.12–1.83) [0.006^*^]	1.54 (0.67–3.57) [0.365]	1.00 (0.99–1.00) [0.066]

The number and ratio of cases in each group were presented as N (%). The average number of cases, average odds ratio, and mean *p*-values for significant results from 1,000 matched samples were calculated. Conditional logistic regression analyses, adjusted for preterm delivery, delivery mode, multiple gestation and ANC duration, were performed for the diseases showing more than 900 significant results in the univariable analyses

^*^Significance at *p*-value < 0.05

*CI* Confidence interval

In study A, among the probable risk factors, the disease with the highest OR was other diseases of digestive system (K92; adjusted OR: 14.12, 95% CI: 2.70–82.26), which was present in 0.20% of all matched cases, and the incidence of leiomyoma of the uterus was the highest (D25; adjusted OR: 3.22, 95% CI: 1.59–6.52), accounting for 1.14% of all matched cases. Among the probable protective factors, gastritis and duodenitis had the lowest OR (K29; adjusted OR: 0.39, 95% CI: 0.22–0.69), accounting for 8.43% of all matched cases, and the incidence of vasomotor and allergic rhinitis was the highest (J30; adjusted OR: 0.58, 95% CI: 0.37–0.92), which accounted for 9.46% of all matched cases (Fig. 2a).

In study B, the possible risk factor was salpingitis and oophoritis (N70; adjusted OR: 3.33, 95% CI: 1.59–6.94), which accounted for 1.26% of all matched cases. Among the probable protective factors, heartburn had lowest OR (R12; adjusted OR: 0.29, 95% CI: 0.12–0.71), which accounted for 5.29% of all cases, and the incidence of gastritis and duodenitis (K29; adjusted OR: 0.75, 95% CI: 0.58–0.95) was the highest, which accounted for 44.15% of all cases (Fig. 2b).

There was no common risk factor in both studies. However, the common protective factors included vasomotor and allergic rhinitis (J30) and gastritis and duodenitis (K29).

## Discussion

Many diseases were identified as significant disease factors from unmatched samples in Table 3 and 4. Because these results were obtained from the unmatched samples, bias should be considered to interpret results. However, diseases with a genetic factor, such as disorders of glycoprotein metabolism from the unmatched sample of study B, may be attributed to the low incidence of these diseases. Therefore, these rare diseases are needed to be verified in a larger population. Meanwhile, some diseases identified from unmatched samples showed significance in hundreds of matched samples, but less than 900. These were not considered as risk factors in this study but may have a weak association with neonatal jaundice. For example, among the disease with OR > 1, alopecia areata showed significance in more than 700 matched samples of study B, which indicates that it may be a maternal risk factor before ANC. In previous studies, alopecia areata was associated with oxidative stress [31, 32]. A decrease in oxidative stress is associated with low serum bilirubin levels [33, 34].

The risk factors for neonatal jaundice included syphilis, surgical follow-up care, leiomyoma of uterus, and other diseases of the digestive system during ANC. Based on previous studies [35, 36], congenital syphilis increases the risk of neonatal jaundice. According to the significant results of surgical follow-up during pregnancy, the type, purpose, and timing of surgery should be identified to explain the relationship between surgical follow-up and neonatal jaundice, which would be limitation of this study using claims data. Leiomyoma can be an extension of the association between leiomyoma as well as preterm birth and cesarean delivery [37, 38].

Other diseases of the digestive system (K92) include hematemesis, melena, and gastrointestinal hemorrhage. However, considering the medical practice of registering a diagnosis, different diseases of the digestive system were included, and the incidence was low. Hence, the significant was low.

Most protective factors identified in study A were associated with infection and inflammation. Recent studies have shown the inverse association between bilirubin and inflammation [39–41]. Thus, inflammation may be associated with the low bilirubin levels, which could decrease the levels of unconjugated bilirubin transferred to neonates.

The pre-pregnancy maternal disease associated with neonatal jaundice was salpingo-oophoritis. Gastritis, dyspepsia, and heartburn are diagnoses associated with the gastrointestinal system. Notably, the OR value for neonatal jaundice was < 1.

The proportion of neonatal jaundice to total delivery cases was 0.40%–0.47%, which was different from its known incidence (30%–80%) [42–46]. This finding could be attributed to the fact that this study was based on neonatal jaundice recorded in the mothers’ claims data. Although prematurity is a risk factor for neonatal jaundice, there was no significant difference in terms of its occurrence between both groups [4, 12]. Since newborns born prematurely are admitted to the neonatal intensive care unit, the diagnosis of neonatal jaundice is rarely applied to the mother. Therefore, jaundice in premature infants may not have been well reflected in this study. Contrary to a known risk factor for neonatal jaundice, vaginal delivery was less common in the jaundice group [47–49]. One possible reason for that may be the differences in the gut microbiota of newborns according to the type of delivery. Infants born via cesarean section have a lower number of *Bifidobacterium* and *Bacteroides* than infants born via vaginal delivery [50–52]. *Bacteroides* reduces unconjugated bilirubin to urobilinoids [53], and an association between the decreased number of *Bifidobacterium* and the elevated levels of bilirubin has been reported [50]. In terms of gut microbiota, cesarean section can be a potential risk factor for neonatal jaundice. The duration of ANC in the jaundice group of study A was significantly short, thereby indicating differences in pregnancy duration or delayed pregnancy diagnosis. A previous study reported the association between the late recognition of pregnancy and adverse outcomes such as neonatal intensive care admission [54].

Identification of the maternal gestational period or pre-pregnancy disease associated with neonatal jaundice may be helpful in counseling mothers preparing for pregnancy or pregnant mothers. For mothers with risk factors, it can predict jaundice for future babies and provide useful information for things to keep in mind after birth. In addition, neonatal jaundice can be prevented through prevention and management of maternal diseases related to neonatal jaundice.

Integration with research on maternal diseases may lead to the development of prenatal care programs to prevent neonatal jaundice. Moreover, it is thought that a more detailed correlation can be derived if the study is conducted including the maternal medication (especially, folic acid and iron, which are essential to take before and during pregnancy). The possible risk factors of certain child diseases including maternal disorders must be assessed from a long-term perspective. Therefore, follow-up studies with a disease network connecting diseases (from maternal to the child disorders) must be performed to assess the association between neonatal jaundice and other pediatric and maternal diseases.

This is the first study to analyze the association between maternal disease not related to pregnancy and neonatal jaundice. Maternal risk factors suggested in this study, including syphilis, leiomyoma, and salpingo-oophoritis, are differentiated from well-known risk factors for neonatal jaundice, such as diabetes mellitus, and suggest there may be unknown pathophysiology. While machine learning-based studies on the prediction of neonatal jaundice required information of neonates such as total serum bilirubin [55, 56], the risk factors identified from the method used in this study can be evaluated with only maternal history before pregnancy or delivery. Another strength includes a large sample size based on health insurance data that covers almost all citizens.

This study had several limitations associated with the use of claims data. The diagnostic code for insurance claims could be differ from the actual diagnosis [57–59]. To ensure data integrity, although mild, the study included mothers who were insured for diseases other than those associated with pregnancy and delivery. The diagnosis codes used in this study were based on KCD-6, the Korean modified version of ICD-10, and numerous medical conditions, including symptoms, are included in the codes. Therefore, statistically significant diagnosis codes about symptoms obtained in this study, such as dyspepsia and heartburn, do not represent the diagnosis of a specific disease. The details about the surgery what other surgical follow-up care referred could not be identified precisely from claims data, which was also a limitation of this study.

## Conclusions

This study has identified that maternal risk factors for neonatal jaundice were syphilis and leiomyoma during pregnancy, and salpingo-oophoritis before pregnancy, and protective factors were infection and inflammatory diseases, and dyspepsia. This has shown significant information that can be used for risk management and the prediction and prevention of neonatal jaundice before or during pregnancy. Furthermore it is necessary to study not only maternal diseases related to neonatal jaundice, but also studies including maternal medication history and long-term prognosis.

## Data Availability

The datasets generated and/or analyzed during the current study are not publicly available due to the regulation that the National Health Insurance Service–National Sample Cohort (NHIS-NSC) data is not provided directly to researchers and can only be accessed via the NHIS analysis system.
